# Replication of Marek's Disease Virus Is Dependent on Synthesis of *De Novo* Fatty Acid and Prostaglandin E_2_

**DOI:** 10.1128/JVI.00352-19

**Published:** 2019-06-14

**Authors:** Nitish Boodhoo, Nitin Kamble, Benedikt B. Kaufer, Shahriar Behboudi

**Affiliations:** a The Pirbright Institute, Pirbright, Woking, United Kingdom; b Institut für Virologie, Freie Universität Berlin, Berlin, Germany; c Faculty of Health and Medical Sciences, School of Veterinary Medicine, University of Surrey, Guilford, Surrey, United Kingdom; Northwestern University

**Keywords:** fatty acid synthesis, Marek’s disease virus, PGE2, avian viruses, virus replication

## Abstract

Marek’s disease virus (MDV) causes deadly lymphoma and induces an imbalance of the lipid metabolism in infected chickens. Here, we discovered that MDV activates the fatty acid synthesis (FAS) pathway in primary chicken embryo fibroblasts (CEFs). In addition, MDV-infected cells contained high levels of fatty acids and showed increased numbers of lipid droplets (LDs). Chemical inhibitors of the FAS pathway (TOFA and C75) reduced MDV titers by approximately 30-fold. Addition of the downstream metabolites, including malonyl-coenzyme A and palmitic acid, completely restored the inhibitory effects of the FAS inhibitors. Furthermore, we could demonstrate that MDV infection activates the COX-2/prostaglandin E_2_ (PGE_2_) pathway, as evident by increased levels of arachidonic acid, COX-2 expression, and PGE_2_ synthesis. Inhibition of the COX-2/PGE_2_ pathway by chemical inhibitors or knockdown of COX2 using short hairpin RNA reduced MDV titers, suggesting that COX-2 promotes virus replication. Exogenous PGE_2_ completely restored the inhibition of the COX-2/PGE_2_ pathway in MDV replication. Unexpectedly, exogenous PGE_2_ also partially rescued the inhibitory effects of FAS inhibitors on MDV replication, suggesting that there is a link between these two pathways in MDV infection. Taken together, our data demonstrate that the FAS and COX-2/PGE_2_ pathways play an important role in the replication of this deadly pathogen.

**IMPORTANCE** Disturbances of the lipid metabolism in chickens infected with MDV contribute to the pathogenesis of disease. However, the role of lipid metabolism in MDV replication remained unknown. Here, we demonstrate that MDV infection activates FAS and induces LD formation. Moreover, our results demonstrate that MDV replication is highly dependent on the FAS pathway and the downstream metabolites. Finally, our results reveal that MDV also activates the COX-2/PGE_2_ pathway, which supports MDV replication by activating PGE_2_/EP2 and PGE_2_/EP4 signaling pathways.

## Abstract (precis)

Disturbances of the lipid metabolism in chickens infected with MDV contribute to the pathogenesis of disease. However, the role of lipid metabolism in MDV replication remained unknown. Here, we demonstrate that MDV infection activates FAS and induces LD formation. Moreover, our results demonstrate that MDV replication is highly dependent on the FAS pathway and the downstream metabolites. Finally, our results reveal that MDV also activates the COX-2/PGE_2_ pathway, which supports MDV replication by activating PGE_2_/EP2 and PGE_2_/EP4 signaling pathways.

## INTRODUCTION

Marek’s disease virus (MDV) is a highly oncogenic herpesvirus that infects chickens and causes deadly lymphoma. The virus is transmitted to naive chickens via the respiratory tract. Macrophages or dendritic cells subsequently transfer the virus to the major lymphoid organs, where it infects B and T cells (1). The infection of T cell subsets plays a crucial role in MDV pathogenesis, while B cells are dispensable for this process (2). CD4^+^ T cells are the predominant cells for the establishment of MDV latency and can be transformed, resulting in T cell lymphomas. T cells also transport the virus to the feather follicle epithelia that produce infectious virus and shed it into the environment (reviewed in reference 3). MDV has been shown to disturb the lipid metabolism of the infected chickens, as it causes atherosclerotic plaque formation (4). Vaccination prevents the development of these plaques (5). Lipid analysis of the arterial smooth muscles from MDV-infected birds revealed a significant increase in nonesterified fatty acids, cholesterol, cholesterol esters, squalene, phospholipids, and triacylglycerol. Furthermore, excess lipid biosynthesis triggers cellular deposition of lipid droplets in MDV-infected cells (4, 5). Despite these intriguing observations, the role of lipid metabolism in MDV-infected cells remained unknown.

In fatty acid synthesis (FAS), acetyl-coenzyme A (CoA) is converted to malonyl-CoA and subsequently to fatty acids. The first step toward FAS is the conversion of citric acid into acetyl-CoA by direct phosphorylation of ATP-citrate lyase (ACLY). The subsequent committed step involves the conversion of acetyl-CoA into malonyl-CoA by acetyl-CoA carboxylase (ACC). The final step involves committed elongation by utilizing both acetyl-CoA and malonyl-CoA coupled to the multifunctional fatty acid synthase (FASN) to generate fatty acids (6). Dengue virus (DENV) (7), West Nile virus (WNV) (8) and hepatitis C virus (HCV) (9) have been shown to preferentially enhance FASN activity and fatty acid synthesis. Fatty acid can contribute to several key biological functions, such as fatty acid oxidation (FAO), β-oxidation, posttranslational modification of proteins, and generation of very-long-chain fatty acids. Prostaglandin E_2_ (PGE_2_), a potent lipid modulator, is derived from enzymatic activity of inducible COX-2 on arachidonic acid (AA). A direct association between induction of COX-2 activity and enhancement of human cytomegalovirus (HCMV) replication has been reported (10, 11).

Here, we demonstrate that MDV infection activates both FAS and COX-2/PGE_2_ pathways, which are crucial for MDV replication. Interestingly, exogenous malonyl-CoA and palmitic acid completely restore the inhibitory effects of FAS inhibitors on MDV replication, suggesting that the synthetized fatty acids are crucial for MDV replication. Intriguingly, addition of PGE_2_ partially restored the inhibitory effects of FAS inhibitors on MDV replication, indicating that the two pathways are connected. Taken together, our results demonstrate that FAS and PGE_2_ synthesis contribute to MDV replication.

(This article was submitted to an online preprint archive [12].)

## RESULTS

### Increased levels of lipid metabolites in MDV-infected cells.

**FIG 1:**
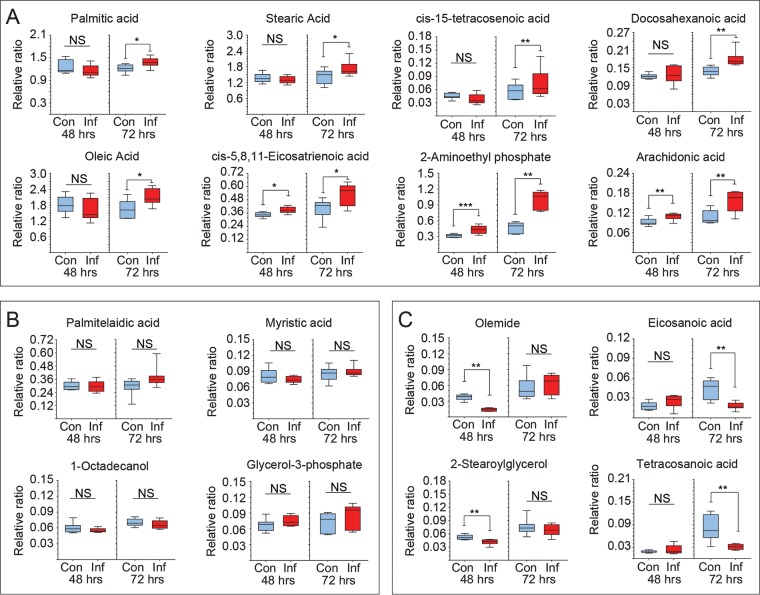
Alteration of fatty acid and arachidonic acid in MDV-infected CEFs. Metabolomics analysis of relative ratio (ratio of area to internal standard) of lipid metabolites from mock (control; Con)- and MDV (RB1B; Inf)-infected CEFs are shown at 48 hpi and 72 hpi. Box and whisker plots show minimum and maximum relative levels of named lipid metabolites that are increased (A), show no change (B), or are decreased (C) in MDV-infected CEFs. Nonparametric Wilcoxon tests (Mann-Whitney) were used to assess normal distribution and test significance, with the results shown as means ± SD. * (*P* = 0.01), ** (*P* = 0.001), and *** (*P* = 0.0005) indicate a statistically significant difference compared to the control. NS, no significant difference. The experiments were performed in biological triplicates with six technical replicates per biological replicate.

Relative production of a panel of metabolites was determined in mock- and MDV-infected primary chicken embryo fibroblasts (CEFs) at 48 and 72 h postinfection (hpi) using two-dimensional gas chromatography with mass spectrometry (GC×GC-MS). The 16 lipid metabolites were detected and analyzed. Eight lipid metabolites, including palmitic acid, were increased at 72 hpi (Fig. 1A), 4 lipid metabolites showed no changes at any time point postinfection (Fig. 1B), and 4 lipid metabolites were decreased (Fig. 1C) at either 48 or 72 hpi. Enhanced levels of fatty acids in the MDV-infected cells can be attributed to increase in FAS and/or breakdown of lipids.

### MDV replication depends on fatty acid synthesis.

**FIG 2:**
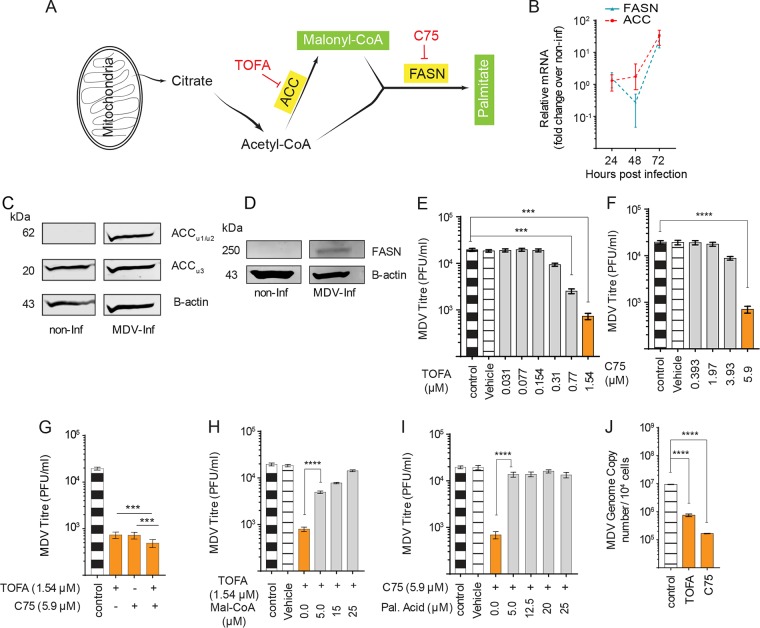
Fatty acid synthesis is involved in MDV replication in CEF cells. (A) Schematic FAS pathways highlighting the relevant pharmacological inhibitors (red) and the respective enzymes (yellow box) as well as metabolites (green box). (B) Fold changes in gene expression in mock- or MDV-infected CEF cells are shown at 24, 48, and 72 hpi. (C and D) Protein levels of ACC (C) and FASN (D) from mock- and MDV-infected CEFs at 72 hpi. (E to G) Alteration in MDV viral titer (PFU/ml) in MDV-infected CEFs treated with TOFA (0.03, 0.077, 0.154, 0.31, 0.77, and 1.54 μM) (E), C75 (0.393, 1.97, 3.93, and 5.9 μM) (F), and TOFA (1.54 μM) (G) in combination with C75 (5.9 μM) at 72 hpi are shown. (H and I) Analysis of MDV viral titer (PFU/ml) in infected CEFs in the presence of malonyl-CoA (30, 25, 15, 10 and 5 μM) with TOFA (1.54 μM) (H) or palmitic acid with C75 (5.9 μM) (I). (J) MDV (RB1B) genome copy numbers per 10^4^ cells (Meq gene with reference ovotransferrin gene) in the presence TOFA (1.54 μM) or C75 (5.9 μM) at 72 hpi. ** (*P* < 0.01), *** (*P* < 0.001), and **** (*P* < 0.0001) indicate a statistically significant difference compared to the control. NS, no significant difference. All viral titer experiments were performed in 6 replicates for plaque assays and 3 replicates for real-time PCR and Western blot assays. The data are representative of 3 independent experiments.

Here, we examined whether MDV infection increases FAS. For a better understanding of FAS, a schematic illustration of the FAS pathway is shown in Fig. 2A. Gene expression of the cellular enzymes involved in the FAS pathway was analyzed in mock- and MDV-infected CEF cells using quantitative PCR (qPCR). The results demonstrate that acetyl-CoA carboxylase (ACC) and fatty acid synthase (FASN) genes were highly upregulated at 72 hpi (Fig. 2B). Analysis of ACC protein levels in mock- and MDV-infected cells was performed using Western blotting. The results demonstrate that the expression of ACC subunit 1 (62 kDa) (Fig. 2C) and FASN proteins (Fig. 2D) is increased in MDV-infected cells. Specific chemical inhibitors for ACC (TOFA) and FASN (C75) were utilized to determine the role of the FAS pathway in MDV replication. Nontoxic concentrations of TOFA, C75, or TOFA and C75 (T/C) were determined based on viability and confluence of the treated CEFs (data not shown). At 72 hpi, viral titers were determined in the presence of the pharmacological inhibitors with addition of the exogenous downstream metabolites (malonyl-CoA and palmitic acid). The inhibitors of ACC (TOFA; 1.54 μM) and FASN (C75; 5.9 μM) significantly reduced MDV titer by 27 (Fig. 2E)- and 28 (Fig. 2F)-fold, respectively. MDV titer was further reduced when the cells were treated with nontoxic concentrations (data not shown) of TOFA (1.54 μM) and C75 (5.9 μM) combined (T/C) compared to those of C75 or TOFA alone (Fig. 2G). Therefore, we used T/C in some experiments to observe maximum inhibitory effects. Addition of malonyl-CoA to the culture in the presence of TOFA restored MDV replication (Fig. 2H). Similarly, palmitic acid restored virus replication in the presence of C75 (Fig. 2I). Treatment of the cells with C75 or TOFA did not reduce plaque sizes (data not shown). To confirm the role of the FAS pathway in MDV replication, we also analyzed MDV genome copy numbers at 72 hpi in the cells treated with TOFA or C75 using qPCR. The results demonstrated that TOFA and C75 also reduce virus copy numbers (Fig. 2J), suggesting that reduction in virus titer by TOFA or C75 is associated with virus copy numbers. Taken together, the results demonstrate that MDV activates the FAS pathway and blocking ACC and FASN decreases MDV titer, which can be rescued by the downstream metabolites.

### MDV infection increases the formation of neutral LDs.

**FIG 3:**
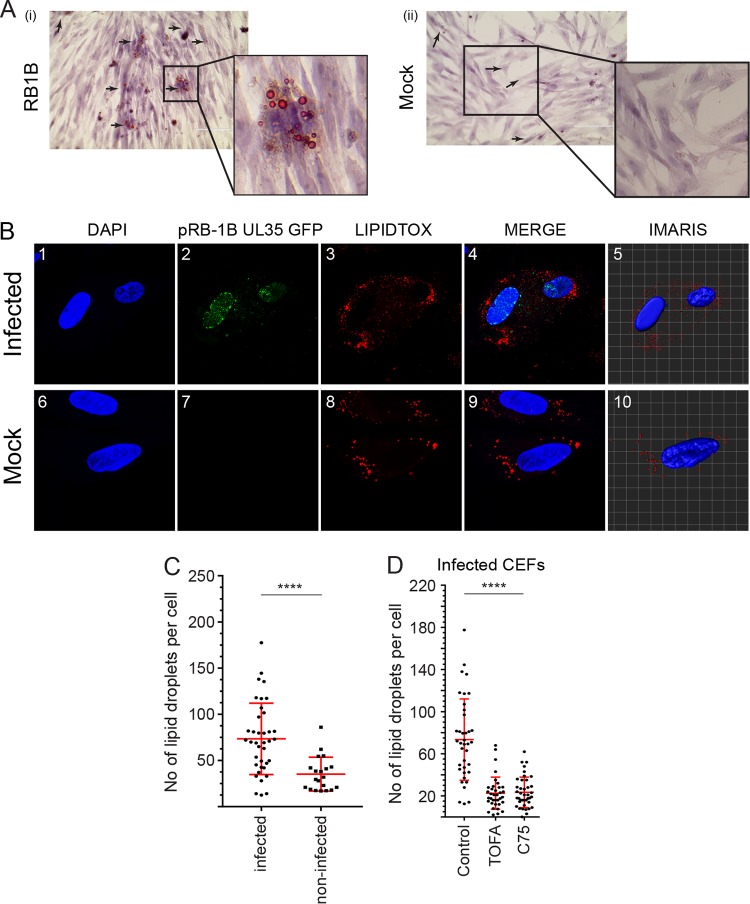
MDV infection increases the formation of neutral lipid droplets. (A) Visualization of cytoplasmic lipids in neutral lipid droplet organelles of CEF cells in MDV-infected (i) and mock-infected (ii) cells (magnification, ×10) at 72 hpi. Black arrows indicate lipid droplets. (B) Confocal microscopy imaging with maximum projection of *z*-stacks for each channel demonstrating nuclear and cytoplasmic distribution of pRB1B UL35-GFP virus (green) and lipid droplets (red). Mock- and pRB1B UL35-GFP-infected cells were fixed at 72 hpi and stained with DAPI (nuclear stain) and the neutral lipid stain LipidTOX-568nm. Images 5 and 10 are three-dimensional representative images analyzed using IMARIS software. *z*-stacks were analyzed using the IMARIS spot function analysis tool to quantify the relative amount of lipid droplets per cell in MDV-infected and mock-infected CEFs (50 to 90 cells). (C) The numbers of lipid droplets per cell in MDV-infected and mock-infected CEFs at 72 hpi. (D) The numbers of lipid droplets per cell in MDV-infected CEFs treated with TOFA (1.54 μM) or C75 (5.9 μM) are shown. ****, statistically significant difference compared to the control (*P* < 0.0001). All experiments were performed in duplicates, and data are representative of 3 independent experiments.

Lipid droplets (LDs) are endoplasmic reticulum (ER)-derived organelles that consist of a neutral lipid core with a phospholipid bilayer, and an increase in the number of LDs is an indication of lipogenesis. Here, the role of FAS on lipid metabolism was evaluated in MDV-infected cells based on LD formation. The presence of lipid droplets was examined in mock- and MDV-infected CEFs using oil red O staining at 72 hpi (Fig. 3A). An increased accumulation of LDs was observed in the MDV-infected cells (Fig. 3Ai) compared to that of the mock-infected cells (Fig. 3Aii). To quantify the number of LDs, pRB1B UL35-GFP-infected CEFs (488 nm) were stained with Red LipidTOX (568 nm), and LD formation was analyzed using confocal microscopy. Larger numbers of LDs per cell were observed in MDV-infected cells than in the mock-infected cells, as determined using the IMARIS software (*P* = 0.0001) (Fig. 3B and C). Treatment of CEFs with TOFA or C75 decreases (*P* = 0.0001) the LD numbers per cell in MDV-infected cells (Fig. 3D). Altogether, these data showed that MDV infection induced the accumulation of LDs and FAS inhibitors reduced LD formation, indicating that there was a direct link between FAS and LD formation in MDV infection.

### MDV activates the COX-2/PGE_2_ pathway.

**FIG 4:**
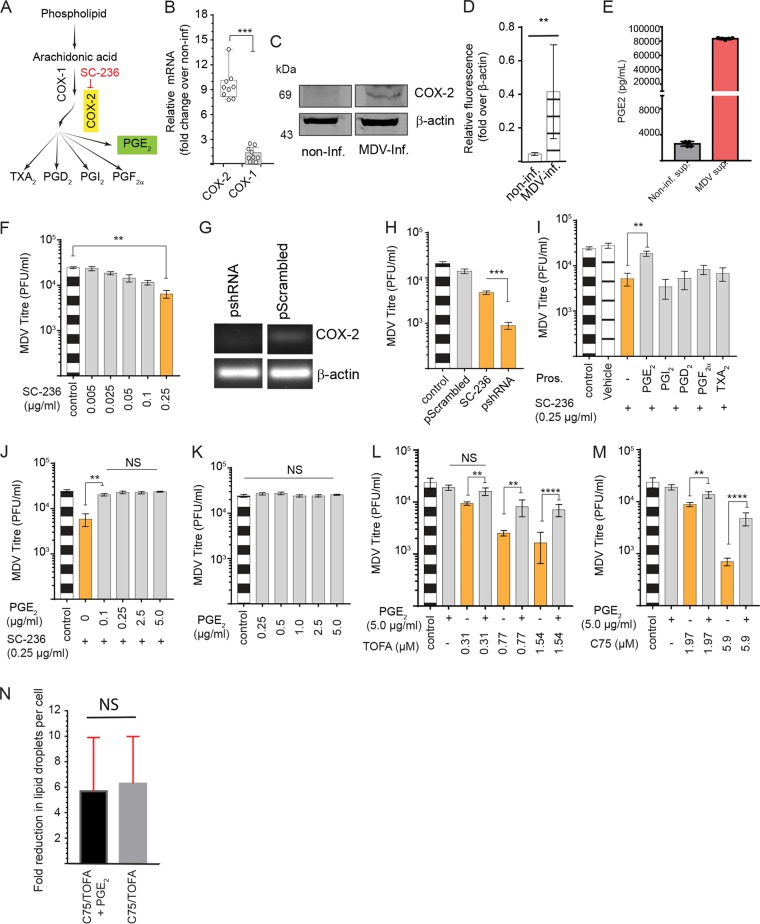
MDV activates COX-2/PGE_2_ pathway. (A) Schematic pathway outlines the relevant pharmacological inhibitor (red) and the respective enzyme (yellow box) as well as the metabolite (green box) studied within the eicosanoid biosynthesis pathway. (B) Fold change in expression of upregulation of COX-2, but not COX-1, gene expression in MDV-infected CEFs at 72 hpi. (C) Western blot analysis showing relative COX-2 protein expression in mock- and MDV-infected CEF cells at 72 hpi. (D) Relative fluorescence representing the expression of COX-2 protein in mock- and MDV-infected cells from five independent experiments at 72 hpi. Levels of β-actin expression were used for normalization. (E) PGE_2_ (pg/ml) concentrations in supernatant of mock- and MDV-infected CEF cells at 72 hpi. (F) MDV titer (PFU/ml) in the MDV-infected CEFs in the presence of a COX-2 inhibitor, SC-236 (0.005, 0.025, 0.05, 0.1, and 0.25 μg/ml). (G) Gel visualization of COX-2 gene silencing by shRNA compared to that of scramble RNA in MDV-infected CEFs. β-Actin was used as an internal control for each reaction. (H) CEFs transfected with shRNA targeting COX-2 silencing or control shRNA (scramble) compared to SC-236 (0.25 μg/ml) at 72 hpi. (I) MDV titer (PFU/ml) in the MDV-infected CEFs treated with SC-236 (0.25 μg/ml) or in combination with PGE_2_ (5.0 μg/ml), PGI_2_ (0.5 μg/ml), PGD_2_ (0.5 μg/ml), PGF_2α_ (0.5 μg/ml), and TXA_2_ (0.5 μg/ml) at 72 hpi. (J and K) MDV titer in the presence of SC-236 (0.25 μg/ml) and different concentrations of PGE_2_ (0.25, 0.5, 1.0, 2.5, and 5.0 μg/ml) (J) or PGE_2_ (0.25, 0.5, 1.0, 2.5, and 5.0 μg/ml) without SC-236 (K) at 72 hpi. (L and M) MDV titer in the presence of different concentrations of TOFA (0.31, 0.77, and 1.54 μM) (L) or C75 (1.97, 3.93, and 5.9 μM) (M) with and without exogenous PGE_2_ (5.0 μg/ml) in the presence of SC-236, T/C, or vehicle at 72 hpi. (N) Fold reduction in numbers of lipid droplets per cell in response to TOFA (1.54 μM) and C75 (5.9 μM) in the presence or absence of exogenous PGE_2_ (5.0 μg/ml) at 72 hpi. * (*P* < 0.01), ** (*P* = 0.0022), *** (*P* = 0.0007), and **** (*P* = 0.0001) indicate statistically significant differences. NS, no statistical difference. Experiments were performed in 6 replicates for plaque assays and 3 replicates for real-time PCR, shRNA silencing, Western blotting, and ELISAs. The data are representative of 3 independent experiments.

LDs are reservoirs of COX-2 and the site of PGE_2_ synthesis (13, 14) by which arachidonic acid (AA) can be converted into eicosanoids, including PGE_2_, thromboxane A_2_ (TXA_2_), prostaglandin D_2_ (PGD_2_), prostaglandin I_2_ (PGI_2_), and prostaglandin F_2_ alpha (PGF_2α_) (Fig. 4A). AA is converted from elongation/desaturation of 18:3 ω-6 fatty acid, an essential fatty acid which can be freed by lipases. In our experiments, we demonstrate that AA levels were elevated in the MDV-infected CEFs (*P* = 0.003) at 48 and 72 hpi (Fig. 1A). No significant difference in COX-1 mRNA transcript expression levels was observed between the mock- and MDV-infected cells at 72 hpi using reverse transcription-PCR (RT-PCR). In contrast, COX-2 mRNA transcripts (Fig. 4B) and protein levels (Fig. 4C) were increased in the MDV-infected cells at 72 hpi. The expression levels of COX-2 in five independent experiments are shown (Fig. 4D). Higher levels of PGE_2_ were detected in the supernatant of MDV-infected cells than in mock-infected cells at 72 hpi using a specific enzyme-linked immunosorbent assay (ELISA) (Fig. 4E). Treatment of the cells with a COX-2 inhibitor, SC-236, reduced MDV titer in a dose-dependent manner (Fig. 4F). To confirm the role of COX-2 in MDV replication, we also demonstrated that short hairpin RNA (shRNA) targeting COX-2 (Fig. 4 G) reduces MDV titers (Fig. 4H). Exogenous PGE_2_, TXA_2_, PGD_2_, PGI_2_, or PGF_2α_ was added to the cells treated with SC-236, and the results demonstrated that PGE_2_ was the only prostanoid that rescued the inhibitory effects of the COX-2 inhibitor on MDV titer (Fig. 4I). Exogenous PGE_2_ (0.1 to 5 μg/ml) rescued the inhibitory effects of SC-236 on MDV titer (Fig. 4J), while it did not alter MDV titer in the absence of the COX-2 inhibitor (Fig. 4K). Strikingly, exogenous PGE_2_ also restored the inhibitory effects of TOFA (Fig. 4L) or C75 (Fig. 4M) on virus titer, suggesting that the inhibitory effect of FAS pathway inhibitors was at least partially dependent on inhibition of PGE_2_ synthesis. Exogenous PGE_2_ recovered an MDV titer in the MDV-infected cells treated with low (0.31 μM), intermediate (0.77 μM), or high (1.54 μM) concentrations of TOFA (Fig. 4L). Similarly, exogenous PGE_2_ rescued the inhibitory effects of low (1.97 μM) and high (5.9 μM) concentrations of C75 on MDV titer (Fig. 4M). To demonstrate that PGE_2_ does not block the function of TOFA and C75 on lipid metabolism, the cells were treated with T/C in the presence or absence of PGE_2_ (5 μg/ml), and the fold reduction in the numbers of lipid droplets per cell were analyzed using confocal microscopy. The results demonstrated that the presence of PGE_2_ did not affect the ability of T/C to reduce lipid droplet numbers (Fig. 4N).

### PGE_2_ supports MDV replication through EP2 and EP4 receptors.

**FIG 5:**
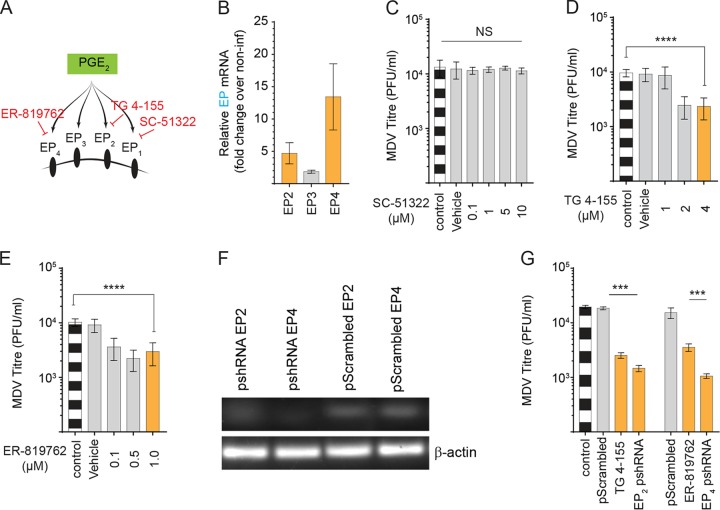
PGE_2_ supports MDV replication through EP2 and EP4 receptors. (A) Schematic pathway outlines the respective metabolites (green box) and the relevant pharmacological inhibitor (red) that were used in this study to block prostaglandin receptors. (B) Fold change in gene expression of PGE_2_ receptors EP2, EP3, and EP4 in MDV-infected CEFs at 72 hpi. (C to E) MDV titer in MDV-infected CEFs treated with different concentrations of SC-51322 (EP1 antagonist; 0.1, 1, 5, and 10 μM) (C), TG 4-155 (EP2 antagonist; 1, 2, and 4 μM) (D), and ER-819762 (EP4 antagonist; 0.1, 0.5, and 1.0 μM) (E) at 72 hpi. (F) Gel visualization of EP2 and EP4 gene silencing by shRNA compared to scramble RNA in MDV-infected CEFs. β-Actin was used as an internal control for each reaction. (G) MDV titer (PFU/ml) in the MDV-infected CEFs transfected with shRNA targeting EP2 and EP4 silencing or control shRNA (scramble) compared to TG 4-155 (4 μM) and ER-819762 (1 μM) at 72 hpi. ****, statistically significant differences (*P* = 0.0001). NS, no statistical difference. Experiments were performed in 6 replicates for plaque assays and 3 replicates for real-time PCR and gene silencing using shRNA. The data are representative of 3 independent experiments.

In mammals, PGE_2_ exerts its biological activities through prostaglandin receptors EP1 to EP4 (Fig. 5A). Chicken EP2, EP3, and EP4 receptors have been cloned and characterized (15, 16), while EP1 receptor has not yet been identified in chickens. Analysis of gene expression in the mock- and MDV-infected CEFs demonstrated that the EP2 and EP4, but not EP3, receptors were upregulated upon MDV infection (Fig. 5B), suggesting that PGE_2_ supported MDV replication through an EP2- and/or EP4-mediated mechanism. Receptor antagonists for EP1 (SC-51322), EP2 (TG 4-155), and EP4 (ER-819762) were utilized to examine the role of different EP receptors in MDV replication *in vitro*. As expected, the EP1 receptor antagonist (SC-51322; 0.1, 1, 5, and 10 μM) had no effect on MDV replication (Fig. 5C). In contrast, TG 4-155 (2 and 4 μM) (Fig. 5D) and ER-819762 (0.1, 0.5 and 1 μM (Fig. 5E) reduced MDV titer (*P* = 0.0001), suggesting that PGE_2_ supports MDV replication through the EP2 and EP4 receptors. To confirm these data, we also used an shRNA system to downregulate the EP2 and EP4 receptors in CEFs (Fig. 4F). The results demonstrate that RNA silencing of EP2 and EP4 by shRNAs and chemical inhibitors (TG 4-155, 4 μM; ER-819762, 1.0 μM) significantly reduced total numbers of MDV plaques (Fig. 5G). Taken together, our data indicate that virus-induced PGE_2_ synthesis supports MDV replication through the EP2 and EP4 receptors.

## DISCUSSION

MDV is an *Alphaherpesvirus* that infects chickens and causes a deadly lymphoproliferative disease. In addition to transformation of CD4^+^ T cells, MDV causes atherosclerosis by disturbing the lipid metabolism in infected chickens (17), which can be inhibited by vaccine-induced immunity (4, 18). MDV is a cell-associated virus, and cell-free virus can only be produced by feather follicle epithelial cells *in vivo*. MDV is transmitted to other chickens via inhalation of virus. No cell-free virus can be generated *in vitro*, and surprisingly, little is known about the mechanism of MDV entry, replication, and egress *in vitro* or *in vivo*. After infection of chickens, MDV can be detected in both immune and nonimmune cells; however, the virus can only be detected in less than 2% of CD4^+^ T cells in the spleen. As there is no specific marker for identification of MDV-transformed T cells (3) and the majority of these cells undergo apoptosis even following *in vitro* T cell activation, which can potentially modulate lipid metabolism, analysis of MDV-induced lipid metabolism in primary lymphoma cells is highly challenging. Similarly, MDV can only be detected in less than 2% of MDV-transformed CD4^+^ T cell lines (3), and lipid metabolism is highly activated in lymphoma cells. Therefore, CEF cells represent more reproducible and reliable cells for studying the role of lipid metabolism in MDV infection in an *in vitro* model. MDV infection can be readily detected in the majority of primary CEF cells, which are the standard cells to investigate the MDV replication cycle *in vitro*. Recently, an *in vitro* infection system for primary B and T cells has been developed that we will also include in future studies (19).

Our analysis of MDV-infected CEFs demonstrates that MDV infection enhances the expression of genes involved in FAS and increases the levels of AA, the prostaglandin precursor. Our data suggest that MDV hijacks host metabolic pathways to provide essential macromolecular synthesis to support infection and replication. *De novo* FAS generates the metabolic intermediate acetyl-CoA, malonyl-CoA, and finally palmitate. Targeted inhibition studies against the enzymes involved in FAS during infection have yielded an alternative understanding of alteration of lipid metabolism in infections with HCMV (20), Epstein-Barr virus (21), and HCV (22, 23). The first step toward FAS is the conversion of citric acid into acetyl-CoA by direct phosphorylation of ACLY. The subsequent step involves the conversion of acetyl-CoA into malonyl-CoA by acetyl-coA carboxylase (ACC) and finally elongation by utilizing both acetyl-CoA and malonyl-CoA coupled to the multifunctional fatty acid synthase (FASN) to make fatty acids. Our results demonstrated that blocking ACC and FASN activity significantly reduces MDV replication, suggesting that MDV preferentially modulates the FAS pathway to generate a variety of lipids that contribute to several key cellular processes. Our data confirm that the inhibitory effects of FAS inhibitors on MDV replication could be overcome by the addition of palmitic acid, a metabolite downstream of FASN in the FAS pathway. This indicates that inhibition of MDV by FAS inhibitors is not simply detrimental to the cell but is essential for the production of infectious virus. There is very limited information on the mechanism involved in alteration of cellular metabolism by viruses. There are only two examples where the viral gene products necessary for changes in each specific metabolic pathway have been identified (reviewed in reference 24). Similarly, there is no information on the mechanism involved in activation of FAS by MDV. Interestingly, MDV encodes a secreted glycoprotein, vLIP, which has homology with lipase but does not have any enzymatic activity (25). Further research is required to determine the role of MDV genes, including vLIP, in activation of lipid metabolism.

**FIG 6:**
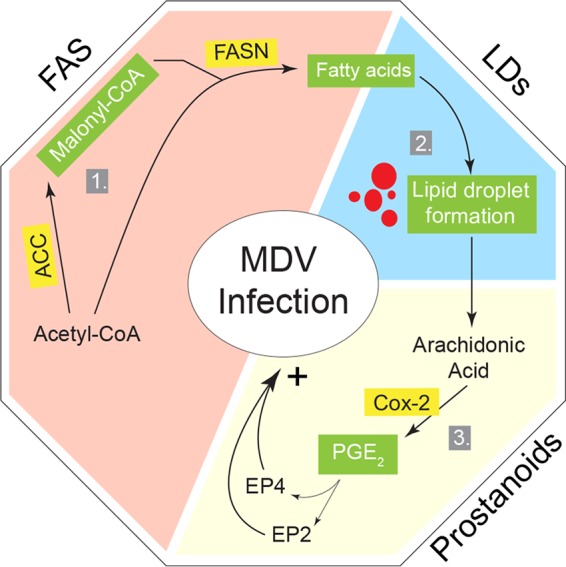
Schematic representation of the model. MDV systematically modulates cellular lipid metabolic pathways to support its replication through FAS and COX-2/PGE_2_ pathways. (1) Infection of CEFs with MDV results in an increase of *de novo* FAS pathway generation, which is required for MDV replication. (2) Fatty acid can be stored in lipid droplet organelles as a site for eicosanoid biosynthesis. (3) Subsequently, PGE_2_ is biosynthesized enzymatically from arachidonic acid by inducible COX-2. Signaling of PGE_2_ is mediated through its EP2 and EP4 receptors in MDV-infected cells. Specific enzymes studied along the pathway and their relative importance for MDV infection are highlighted in yellow. Major metabolites which were identified as essential lipids are highlighted in green.

It is known that fatty acids are essential components for the initiation of key cellular processes, such as membrane lipid synthesis, generation of LDs, and eicosanoid synthesis (26). LDs are classically defined as organelles with stored neutral lipids, and some reports suggest that some viruses promote LD formation, which is involved in virus replication and assembly (27–29). Our data demonstrate that MDV infection also increases LD formation; however, the exact role of LDs in MDV infection is still unknown. LD formation in MDV-infected cells is dependent on the FAS pathway, as FAS inhibitors reduced the numbers of LDs. This organelle is a significant source of triglyceride-derived arachidonic acid (AA), reservoir of COX-2 and site of PGE_2_ synthesis (13, 14, 30). Our results demonstrate that MDV infection increases the levels of FAS, AA, COX-2, and PGE_2_ synthesis; however, further studies are required to establish whether PGE_2_ synthesis occurs in LDs of MDV-infected cells. Furthermore, our results confirm previous observations on the role of FAS in LD formation, replication of pathogens, and PGE_2_ synthesis (13). In MDV-infected cells, both metabolic and nonmetabolic factors could be involved in COX-2 activation. Upregulation of COX-2 in the MDV-infected cells could be induced by several mechanisms, including transforming growth factor beta (TGF-β) (31, 32), cyclic AMP (33), and activation of NF-κB (34). Further studies are required to support the role of these factors in the activation of COX-2 during MDV infection. COX-2-inducing factors such as TGF-β (32), which also activates fatty acid synthesis (31, 35), might be involved in activation of COX-2 in MDV-infected cells. In fact, we have recently shown that MDV infection increases the induction of TGF-β *in vivo* (36); however, further studies are required to examine the role of MDV-induced TGF-β in the induction of COX-2 during MDV infection. It has been shown that COX-2 in LDs, but not in cytoplasm, is involved in PGE_2_ synthesis following infection or activation of the cells (14). Further experiments are required to establish the role of FAS-induced LDs in the activation of the COX-2/PGE_2_ pathway by MDV. Finally, we demonstrated that PGE_2_ promotes MDV replication via EP2 and EP4 receptors, which are upregulated in MDV-infected cells. PGE_2_ can contribute to virus replication by inhibition of nitric oxide production (37), type I interferon production (38), and modulation of immune cells, including antigen-presenting cells and T cells (39). We have recently reported that soluble factors released from MDV-transformed T cells inhibit the function of chicken T cells in a COX-2-dependent manner (36). The proposed model for the role of FAS and COX-2/PGE_2_ pathways in MDV infection is summarized in Fig. 6. This study has assisted us in the identification of potential pathways involved in MDV pathogenesis and may eventually lead to the development of control measures. However, we do not anticipate the use of FAS inhibitors as an antiviral agent in poultry because of their systemic side effects and potential residue in meat and eggs. Taken together, our results demonstrate that MDV activates FAS and COX-2/PGE_2_ pathways, and replication of MDV is dependent on PGE_2_ synthesis, which supports MDV infectivity through EP2 and EP4 receptors.

## MATERIALS AND METHODS

### Ethics statement.

Ten-day-old specific-pathogen-free embryonated chicken eggs (Valo Biomedia GmbH) were used to generate primary chicken embryonic fibroblast cells (CEFs). All embryonated chicken eggs were handled in accordance with the guidance and regulations of Europe and the United Kingdom Home Office under project license number 30/3169. As part of this process, the work has undergone scrutiny and approval by the ethics committee at The Pirbright Institute.

### Chemicals and antibodies.

Chemicals used include SB 204990 (Thermo Fisher Scientific, Paisley, UK), TOFA, C75, clofibrate, palmitic acid, and SC-236 (Sigma-Aldrich, Dorset, UK), PGD_2_, PGI_2_, and PGE_2_ (Cambridge Bioscience, Cambridge, UK), and SC-51322, TG-4-155, and ER-819762 (Bio-Techne Ltd., Abingdon, UK), and all were reconstituted in dimethyl sulfoxide. TXA_2_ (Cambridge Bioscience, Cambridge, UK) was reconstitute in ethanol. PGF_2α_ (Cambridge Bioscience, Cambridge, UK) and etomoxir and malonyl-CoA (Sigma-Aldrich, Dorset, UK) were reconstituted in E199 medium. Anti-β-actin mouse monoclonal antibody (MAb), anti-FASN goat polyclonal antibody (pAb), anti-COX2 goat pAb, and anti-ACC rabbit MAb (Abcam, Cambridge, UK) were used for Western blot analysis. Donkey pAb to goat IgG, goat pAb to mouse IgG, and goat pAb to rabbit IgG were also purchased from Abcam, Cambridge, UK.

### CEF culture and plaque assay.

CEFs were seeded at a rate of 1.5 × 10^5^ cells/ml in 24-well plates in E199 medium supplemented with 5% fetal calf serum (FCS), and virus titer was determined 72 h postinfection with cell-associated MDV in the presence or absence of the inhibitors. To identify nontoxic concentrations of the chemicals, mock- and MDV-infected CEFs were exposed to the chemicals or vehicles, cell morphology and adherence/confluence were monitored under light microscopy, and then cells were stained with 7-AAD (BD Bioscience, Oxford, UK) and acquired using MACSQuant flow cytometry and FlowJo software for analysis of the data. Nontoxic concentrations of the inhibitors and chemicals were selected based on flow cytometry data and confluence. MDV-infected cells were titrated onto fresh CEFs, and 72 h postinfection, the cells were fixed and incubated with mouse anti-gB MAb (clone HB-3), as previously described (40), followed by horseradish peroxidase-conjugated rabbit anti-mouse Ig. After development of the plaques using AEC substrate, the cells were washed with super Q water and viral plaques were counted using a light microscope.

### Metabolomics.

CEFs were either mock infected or infected with the RB1B strain of MDV (100 PFU per 1.5 × 10^5^ cells) in triplicates and harvested at 48 and 72 h postinfection (hpi). The levels of metabolites were determined using GC×GC-MS (Target Discovery Institute, University of Oxford), and the data were analyzed as described previously (41). Those metabolites for which we had high confidence in their identifications are shown. The lipid profiles of mock- and MDV-infected cells were analyzed in biological triplicates with up to six technical replicates per biological replicate. The data were adjusted and normalized based on protein content. Virus infection did not change the size of the cells, as determined by microscopy.

### Knockdown of EP2, EP4, and COX-2 gene expression.

Small short hairpin RNAs (shRNAs) and control scrambled RNAs were designed using short interfering RNA (siRNA) Wizard software, and these were chemically synthesized (InvivoGene, Toulouse, France) (Table 1). Construction of the shRNA harboring plasmid was performed per the manufacturer’s instructions. For silencing purpose, CEFs were transfected with the silencing and scrambled plasmid backbone containing green fluorescent protein using Lipofectamine stem reagent (Life Technologies, Warrington, UK). The transfection efficiency was observed at 24 h posttransfection using fluorescence microscopy, and cells showing more than 70% transfection efficiency were used for MDV infection.

**TABLE 1:** siRNA sequences against COX-2, EP2, and EP4

Gene and target	Oligonucleotide sequence^*a*^
COX-2(PTGER2)	
siRNA 1	5′-**ACCTC**GATTGACAGCCCACCAACATA**TCAAGAG**TATGTTGGTGGGCTGTCAATC**TT**-3′
	5′-**CAAAAA**GATTGACAGCCCACCAACATA**CTCTTGA**TATGTTGGTGGGCTGTCAATCG-3′
Scrambled 1	5′-**ACCTC**GTGCCCAACACGCAATTAACA**TCAAGAG**TGTTAATTGCGTGTTGGGCAC**TT**-3′
	5′-**CAAAAA**GTGCCCAACACGCAATTAACA**CTCTTGA**TGTTAATTGCGTGTTGGGCAC**G**-3′
siRNA 2	5′-**ACCTC**GTGGGATGATGAGCAGCTATT**TCAAGAG**AATAGCTGCTCATCATCCCAC**TT**-3′
	5′-**CAAAAA**GTGGGATGATGAGCAGCTATT**CTCTTGA**AATAGCTGCTCATCATCCCAC**G**-3′
Scrambled 2	5′-**ACCTC**GCTGTGAGTGCGAAGAATTGT**TCAAGAG**ACAATTCTTCGCACTCACAGC**TT**-3′
	5′-**CAAAAA**GCTGTGAGTGCGAAGAATTGT**CTCTTGA**ACAATTCTTCGCACTCACAGC**G**-3′
EP2 (PTGER2)	
siRNA 1	5′-**ACCTC**GCGCCTACGTCAACAAGTTCA**TCAAGAG**TGAACTTGTTGACGTAGGCGC**TT**-3′
	5′-**CAAAAA**GCGCCTACGTCAACAAGTTCA**CTCTTGA**TGAACTTGTTGACGTAGGCGC**G**-3′
Scrambled 1	5′-**ACCTC**GGCCGAACTAAGCCCACTTTA**TCAAGAG**TAAAGTGGGCTTAGTTCGGCC**TT**-3′
	5′-**CAAAAA**GGCCGAACTAAGCCCACTTTA**CTCTTGA**TAAAGTGGGCTTAGTTCGGCC**G**-3′
EP4 (PTGER4)	
siRNA 1	5′-**ACCTC**GCTCCTTCATCCTCCTCTTCTT**CAAGAG**AGAAGAGGAGGATGAAGGAGC**TT**-3′
	5′-**CAAAAA**GCTCCTTCATCCTCCTCTTCT**CTCTTGA**AGAAGAGGAGGATGAAGGAGC**G**-3′
Scrambled 1	5′-**ACCTC**GCTCCTCTCCCCTTCTCTATTT**CAAGAG**AATAGAGAAGGGGAGAGGAGC**TT**-3′
	5′-**CAAAAA**GCTCCTCTCCCCTTCTCTATT**CTCTTGA**AATAGAGAAGGGGAGAGGAGC**G**-3′

a The boldface at 5' ends represents sequences for duplex stability, which provide resistance to RNase degradation. Boldface in the middle of a segment represents the site for hairpin loop folding. Boldface at the 3' region represent oligonucleotide overhangs, which are resistant to nuclease degradation.

### qPCR to amplify MDV genes.

DNA samples were isolated from 5 × 10^6^ cells using the DNeasy-96 kit (Qiagen, Manchester, UK) according to the manufacturer’s instructions. A master mix was prepared containing primers Meq-FP and Meq-RP (0.4 μM), Meq probes (0.2 μM), *ovo* forward and reverse primers (0.4 μM), and *ovo* probe (0.2 μM; 5′Yakima Yellow-3′TAMRA; Eurogentec) and ABsolute Blue qPCR, low Rox, master mix (Thermo Fisher Scientific, Paisley, UK). A standard curve generated for both Meq (10-fold serial dilutions prepared from plasmid construct with Meq target) and the *ovo* gene (10-fold serial dilutions prepared from plasmid construct with *ovo* target) were used to normalize DNA samples and to quantify MDV genomes per 10^4^ cells. All reactions were performed in triplicates to detect both Meq and the chicken ovotransferrin (*ovo*) gene on an ABI7500 system (Applied Biosystems) using standard conditions. MDV genomes were normalized and are reported as viral genome per 10^4^ cells.

### Real-Time PCR.

Total RNA was extracted from mock- and MDV-infected CEFs using TRIzol (Thermo Fisher Scientific, Paisley, UK) according to the manufacturer’s protocol and treated with DNA-free DNase. Subsequently, 1 μg of purified RNA was reverse transcribed to cDNA using a Superscript III first-strand synthesis kit (Thermo Fisher Scientific, Paisley, UK) and oligo(dT) primers according to the manufacturer’s recommended protocol. The resulting cDNA was diluted 1 : 10 in diethyl pyrocarbonate-treated water. Quantitative real-time PCR using SYBR green was performed on diluted cDNA using a LightCycler 480 II (Roche Diagnostics GmbH, Mannheim, GER) as described previously (36). Data represent means from 6 biological replicates. The fold decreases in the mRNA transcript for EP2, EP4, and COX-2 genes were detected by one-step RT-PCR performed with a Luna Universal one-step RT-qPCR kit (NEB) on an Applied Biosystems 7500 qPCR system using primers outlined in Table 2.

**TABLE 2:** List of primers used for RT-PCR

Gene name	Accession no.	Primer and sequence	*T_m_*^*a*^ (^o^C)	Product size (bp)
Acetyl-CoA carboxylase (ACC)	J03541.1	Fwd, ACGTTCGAAGGGCGTACATT	60	161
		Rev, TACGTGGACCATCCCGTAGT		
Fatty acid synthase (FASN)	J03860.1	Fwd, CTTTGGTGGTTCGAGGTGGT	60	170
		Rev, CTGTGGGAACCTTGCTTGGA		
		Rev, AATATCCGCTCCAATGCCTCC		
Prostaglandin receptor 2 (EP2)	NM_001083365.1	Fwd, CCTTCACGATCTGCGCCTAC	60	92
		Rev, GGGGTTGATGGAGAGGAAGC		
Prostaglandin receptor 3 (EP3)	NM_001040468.1	Fwd, GCTGCTGGTAACGATGCTGA	60	177
		Rev, CGGAGCAGCAGATAAACCCA		
Prostaglandin receptor 4 (EP4)	NM_001081503.1	Fwd, ATGTTCCAGGGTACAGGTTTTGT	60	175
		Rev, GCCTAGCCTGCACGGTGTT		
Clyclooxygenase 1 (COX-1)	XM_425326	Fwd, TCAGGTGGTTCTGGGACATCA	60	123
		Rev, TGTAGCCGTACTGGGAGTTGAA		
Clyclooxygenase 2 (COX-2)	NM_001167718	Fwd, CTGCTCCCTCCCATGTCAGA	60	123
		Rev, CACGTGAAGAATTCCGGTGTT		
Cytoplasmic beta actin	X00182	Fwd, TGCTGTGTTCCCATCTATCG	60	150
		Rev, TTGGTGACAATACCGTGTTCA		

a *T_m_*, melting temperature.

### Western blotting.

Mock- and MDV-infected CEFs were lysed in radioimmunoprecipitation assay buffer and quantified by spectrophotometry. The lysate was suspended in the Laemmli’s sample loading buffer (Sigma-Aldrich, Dorset, UK) and loaded in 10% SDS-PAGE. After semidry transfer of SDS-PAGE on a nitrocellulose membrane, the membrane was blocked in 5% skim milk powder for 2 h. The membranes were incubated with primary antibody for 12 h at 4°C, washed, and incubated with secondary antibodies. Finally, the blots were probed with the Odyssey CLx imaging system (Li-Cor, USA), and bands were quantified with Image Studio Lite software.

### Prostaglandin E_2_ ELISA.

PGE_2_ was quantified in supernatant of mock- and MDV-infected cells using a colorimetric assay (R&D Systems, Abingdon, UK) based on competition between unlabeled PGE_2_ in the sample and a fixed amount of conjugated PGE_2_. The assay was performed according to the recommendations of the assay kit manufacturer. In brief, CEFs were either mock infected or infected with RB1B, and the levels of PGE_2_ in the supernatant were determined at 72 hpi or the assay results were measured using optical density at 450 nm. The concentrations of PGE_2_ were determined against a standard curve.

### Oil red O staining.

For analysis of lipid droplets, cell monolayer was washed with phosphate-buffered saline (PBS) and fixed with 4% formaldehyde for 30 min. Cells were subsequently stained with oil red O solution for 30 min, followed by a wash in PBS. Cells were counterstained with hematoxylin for 3 min, followed by a wash with super Q water. Plates were visualized and imaged using a light microscope, and the pictures were processed using Adobe Photoshop software.

### Fluorescence confocal microscopy.

CEFs were seeded in 24-well plates that contained 12-mm-diameter round coverslips at a rate of 1.0 × 10^5^ cells per well. At 72 h after mock infection or infection with the pRB1B UL35-GFP virus (the virus was kindly provided by V. K. Nair, The Pirbright Institute), the samples were prepared for imaging. In brief, mock- or MDV-infected CEFs were fixed with 4% formaldehyde for 30 min and incubated with HCS LipidTOX Red neutral lipid stain, and then nuclei were labeled with 4′,6-diamidino-2-phenylindole (DAPI). Cells were viewed using a Leica SP2 laser-scanning confocal microscope. The data are presented as maximum projections of *z*-stacks (23 to 25 sections; spacing, 0.3 mm), which were analyzed using IMARIS (Bitplane Scientific Software). Ninety MDV-infected cells and 40 mock-infected cells were analyzed, and their LipidTOX-labeled neutral lipid-containing organelles were detected with the spot function of IMARIS. Images were processed using Adobe Photoshop software.

### Statistical analysis.

All data are presented as means ± standard deviations (SD) from at least three independent experiments. Quantification was performed using Graph Pad Prism 7 for Windows. The differences between groups, in each experiment, were analyzed by nonparametric Wilcoxon tests (Mann-Whitney) or by Kruskal-Wallis test (one-way analysis of variance, nonparametric). Results were considered statistically significant at a *P *value of *<*0.05 (*).
